# Observations on Amenorrhœa

**Published:** 1818-11

**Authors:** J. Mahon


*For the London Medical and Physical Journal.*

### *Observations on Amenorrhœa.* By J. MAHON, M.D.

THERE is no species of disease, or rather effect of disease, respecting which less practical information can be derived from books, than that designated by the above term. Systematic writers have dismissed it with a few general observations; and, to my knowledge, it has not, at least in this country, formed the express subject of a work.

As the various symptoms which ensue from this state of disease are in the highest degree perplexing to the young practitioner, and for which reflection without experience, or observations deduced from experience, can render him so little assistance in supplying indications for the mode of treatment, I have considered that I might render some service to that part of the members of the medical profession, by collecting together some observations deduced from rather extensive practice in this species of disease. Should my expectations prove unfounded, I shall, at least, have the gratification of reflecting that I have, in the retired as well as in the active part of my life, used my endeavours to improve and extend the benefits of the medical art.

I shall confine my remarks, at present, to those conditions of the system under which absence of the menstrual evacuation takes place after it has been once established; as to treat of the causes of its primary non-appearance, would lead to discussion too extensive to be embraced within the limits of a publication like your Journal.

There is no class of functions more intimately connected with the mind, both in receiving from, and transmitting to it, impressions, than that of the generative system; and, although not immediately connected by a correspondence of intention with the general functions of nutrition and excretion, it is allied with them by sympathy in a peculiar manner. These circumstances must, therefore, be taken into consideration, as well as the general physical conditions of the system, in order to acquire a knowledge of the nature of amenorrhœa.

The states of body under which amenorrhœa most frequently occurs are—an *exsangeous* and *debilitated habit*, and that which has been described by writers as constituting the *nervous temperament*. It occasionally ensues from too great *plethora*, when it has been productive of local or general excitement.

The derangement of the functions of an organ, which ensues from the abstraction of its natural stimulus, must also be considered. We have daily occasion to witness the effects of that law of nature in animal bodies, by which the actions of a part are rendered vigorous by exertion, and languish or become lost by long-continued repose. That the functions of the uterus will be influenced, from the effect of this law, by our social regulations and habits, is evident. We thus find that suppression of the menses, as a disease, most frequently affects *unmarried* females. The usual mode of education also now adopted, particularly of those of the higher ranks, and their subsequent habits, all of which tend to increase sensibility and diminish corporeal force, contribute, in an eminent degree, to procure that deranged state of the uterine system we so frequently witness.

There are many occasional causes, which must also be noticed; such as long-continued powerful mental impressions, of whatever kind, by which the vital powers are particularly directed to the brain, or other organs than the uterus; the debilitating passions in general; shocks to the nervous system; great loss of blood, accidental or intentional; spare and innutritious diet; the derangement of action consequent on the sudden application of cold to the body; and, in short, all causes which subvert the due and regular action of the animal economy in general.

The symptoms arising from amenorrhœa may be divided into three classes,—immediately local, general, and subsequently local. The most usual of the former are pains, and a sense of distention about the uterus, and a mucous discharge from the vagina; and sometimes chronic inflammation of the uterus and its appendages, more particularly of the ovaries, terminating in dropsy. The general symptoms are numerous and variable, being influenced both by the constitution of the patient, and the length of time which the disease may have existed. The usual are fever, congestion or inflammation of various parts, hæmorrhage, a variety of affections usually termed nervous, and a disordered state of the digestive organs. Many of the above are local symptoms; but they are so intimately connected with *general* disorder, that I distinguish them from those I would term, strictly, the *subsequently local symptoms.* These are organic diseases of various parts, frequently terminating in change of structure; as tubercles in the lungs, obstruction of the mesenteric glands, slow enlargement of the liver, cancer, &c.; all of which may, perhaps, be attributed to a predisposition in the parts, acted on by amenorrhœa, as an exciting cause.

Inflammatory fever attacks the young and vigorous, and, if suffered to run on uninterruptedly, often affects its cure, by re-establishing the menstrual evacuation; but it is also frequently accompanied with inflammation of the uterus, or some other organ, which does not terminate in so favourable a manner. Fever of a remittent type, or sometimes simulating the hectic character, generally affects those of a more spare and irritable habit, and usually continues till the cause be removed; but sometimes gives place to other disorders. General inflammation of the serous membranes occasionally happens, terminating in dropsy, if bleeding and purging be not employed. Inflammation of the uterus sometimes appears to arise from immediate irritation of that organ; at others, from the general plethoric state of the system.

Spontaneous hæmorrhage usually takes place from the stomach or nostrils, and is then, in general, to be considered a favourable occurrence; when it comes from the lungs, it is rather an alarming symptom; for, although it frequently occurs repeatedly, for a considerable length of time, without being productive of any serious effects, yet it occasionally lays the foundation of phthisis.

The most distressing symptoms are those nervous affections which are referred to various parts of the body, and which appear to arise from sympathy with the uterus; such as hysterics, St. Vitus's dance, spasmodic asthma, &c.: these often continue after the cause of them is removed.

Disorders of the stomach and bowels evince themselves by all the symptoms of indigestion, with depraved appetite, costiveness and purging alternately, with diseased secretion from the colon and large intestines generally: these complaints, though sometimes the consequence, are also very commonly the cause of amenorrhœa. Persons of debilitated habits are usually the subjects of them.

Organic diseases, of the kind before mentioned, most usually affect parts which may have had a predisposition to disease: they are with difficulty removed, unless the return of the menses be quickly effected.

In the *treatment* of amenorrhœa and its symptoms, we must consider the state of the general system which influenced the production of that disease as the primary cause; and the stoppage of the natural evacuation, and the symptoms which have ensued, as merely first and secondary trains of effects. Much mischief has been done by directing medical efforts to the reproduction of the secretion of the uterus alone, considering the absence of which as the cause of all the disorder witnessed. When it has arisen merely from occasional causes, such measures may be effectual; but, when it arises from general derangement, they are entirely useless. On the general health being restored, the secretion will usually return; or, if not, endeavours may then be made to produce it, by agents which particularly affect the uterus. The consequences which ensue from amenorrhœa may generally be temporarily removed, although the cause remain; and this is, in the greater number of instances, to be effected by purging and the abstraction of blood.

When amenorrhœa arises from a debilitated and exsangeous state of the system, it does not in itself merit attention; but if this state of exhaustion be the consequence of previous excess of action, and the suppression of long continuance, the secretion is sometimes never restored, although the health may be re-established. The uterus, in these cases, appears to have lost all disposition for the functions for which it was intended by nature. The same thing sometimes ensues from inflammation of the uterus. We have remarked, in some cases of this kind, that the woman has lost somewhat of the female character.

In our endeavours to remove the symptoms of amenorrhœa, we must consider the greater or less violence of them, and the length of time they may have existed. When it is produced by sudden causes,—as cold, violent passions and emotions of the mind, &c. the principal indication will be to removes the febrile excitement, if such be induced, quiet the general constitutional disturbance, and prevent the inconvenience that might ensue from the want of the accustomed evacuation. The most effectual means for these purposes will be the exhibition of purgative and diaphoretic medicines, the warm bath, and in most cases the abstraction of blood. Should there be no apparent disposition to the return of the evacuation at the ensuing period, the medicines termed emenagogues may be given ; and, when no morbid excitement is present, electricity may be employed. The hysterical affections which sometimes ensue will be most effectually relieved by an emetic, followed by a full dose of opium. The bowels should be kept in rather a lax state.

Disorders of the stomach, hæmorrhage, and the organic diseases which may ensue, must be treated from the same indications as when they arise from general causes.

Cutaneous diseases of an anomalous character sometimes appear. They must be considered as symptoms for which no efforts directed to their immediate removal are required. The warm bath may, however, be sometimes advisable.

In cases of suppression of long continuance, the indication is to remedy the general disorder of the system, to which it is to be attributed; and, after that, to solicit the return of the functions of the uterus by gentle stimulants. The means for effecting the former must be regulated by the peculiar state of the constitution.

In persons of a plethoric habit, abstraction of blood from the arm, when any organ, particularly the uterus, is the seat of chronic inflammation; local evacuation of the same kind, by leeches or cupping-glasses, should be employed. This may be conjoined with the use of antimonials and purgatives, and particularly with the warm bath. When there is not much excitement of the system, general blood-letting may be omitted. There is frequently in this state, pain and a forcing sensation about the uterus, which occurs periodically: the application of from twelve to twenty leeches to the region of the uterus should be resorted to at these times. Amenorrhœa accompanying a plethoric state of the system, it must be observed, perhaps always depends on general or local excitement disturbing the regular functions of the uterus: such causes should always be expected, and of course carefully sought after, as it is on the removal of them that this effect is to be obverted. It will be unnecessary to dwell on this point, the mode of treatment is so obvious.

Those of a nervous habit rarely require the use of general blood-letting, although the local employment of it may sometimes be advisable, as congestion of blood in the uterus is very likely to happen. The general physical and moral habits of patients should here be particularly attended to. They should respire a pure and cool air; the clothing should be light; active exercise, particularly walking, will be highly serviceable; and, when it can be effected, travelling in a pleasant country will be advisable. The diet must be rather spare, but composed of a considerable proportion of animal food. Anxiety of mind should be avoided, and indeed all powerful mental emotions. A peculiar susceptibility of nervous irritation, both from external and sympathetic impressions, is sometimes conjoined with a plethoric state of the system. Patients in this state are very liable to hysterical and other convulsive disorders: here the lancet must not be spared. There is generally a peculiar determination of blood to the uterus in these cases, which, when unrelieved, frequently terminates in chronic inflammation of that organ. A very spare diet, and daily exercise so as to produce fatigue, are here particularly advisable. The occasional use of emetics, and the tepid bath, will be very beneficial. This frequently depends on moral causes. Though irregularity, rather than suppression, of the menses is more frequently produced in these instances. The general indications for the mode of treatment are here obvious.

A defect of sensibility of the uterine system requires the use of the same means as those which are adopted for the complaint when it arises from a weak and torpid state of the body generally. In these cases, electricity has been productive of the most prompt and beneficial effects. It is here that tonic medicines, as steel and bark, and the cold bath, have been productive of so much advantage as to occasion their adoption too frequently in the treatment of amenorrhœa. There are but few cases, indeed, to which they are really applicable. The class of medicines usually termed emenagogues, the best of which is black hellebore, must be confined to this state of the disease, and must here only be employed in conjunction with the measures for the improvement of the general habit.

I would desire to impress this observation on the minds of the younger part of my readers—that amenorrhœa is to be treated as the consequence, not the cause, of disease.

The above is offered as a mere sketch; but, if it will serve as a guide to direct the observations of others, my object will be accomplished.

*Bandon-hill, Cork; Oct.* 3, 1818.

